# Supplementary material for: Functional reorganization of motor cortex connectivity during learning
Source: bioRxiv. 2026 Mar 5:2026.03.03.709199. Preprint. [Version 1] doi: 10.64898/2026.03.03.709199 (PMC12991118; doi:10.64898/2026.03.03.709199)
Supplement: Supplement 1 [file NIHPP2026.03.03.709199v1-supplement-1.pdf]

bioRxiv preprint doi: https://doi.org/10.64898/2026.03.03.709199; this version posted March 5, 2026. The copyright holder for this preprint (which was not certified by peer review) is the author/funder, who has granted bioRxiv a license to display the preprint in perpetuity. It is made available under aCC-BY 4.0 International license.

Supplemental Figures

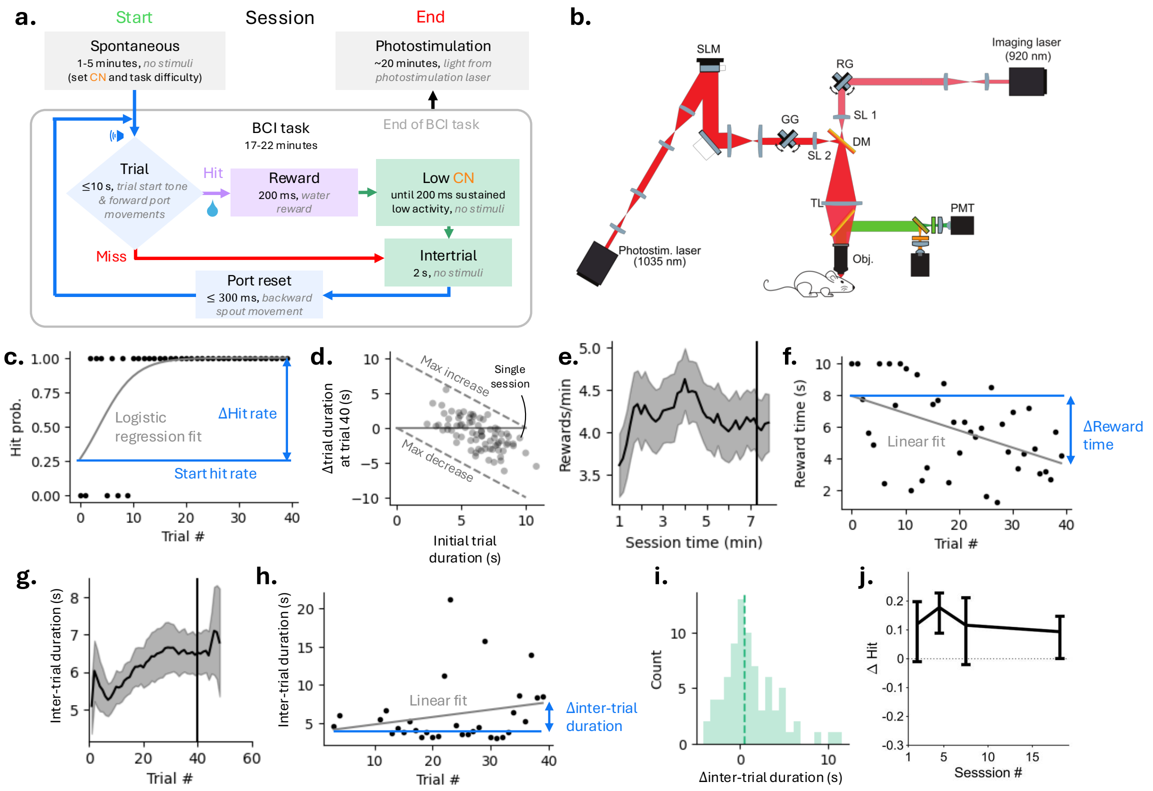

Figure S1: Supplement to Figure 1. **(a)** Schematic of BCI trial structure. **(b)** Schematic of microscope. **(c)** Exemplar logistic regression fit on hit trial probability versus trial number, over first 40 trials. Same exemplar session shown in Fig. 1. Change in hit rate defined as difference in fit over first 40 trials. **(d)** Change in trial duration versus starting trial duration for all sessions. Dotted diagonal lines show maximum and minimum possible trial duration improvement given starting trial duration. **(e)** Rewards per minute versus session time (mean over sessions, 95% bootstrap shading). Black line shows median time to 40 trials. **(f)** Linear regression fit to time to reward (from trial start) versus trial number for an exemplar session, over first 40 trials. Same exemplar session shown in Fig. 1. Change in reward time defined as difference in fit over first 40 trials. **(g)** Inter-trial duration, the time to next trial start after reward time, versus trial number (mean over sessions, 95% bootstrap shading). **(h)** Exemplar linear regression fit to inter-trial duration versus trial number for an exemplar session, over first 40 trials. Same exemplar session shown in Fig. 1. **(i)** Histogram of change in inter-trial durations over first 40 trials, dotted line shows median across sessions. **(j)** BCI performance (Δhit rate) versus number of sessions, showing negligible task meta-learning (mean, error bars bootstrapped 95% CI).

bioRxiv preprint doi: https://doi.org/10.64898/2026.03.03.709199; this version posted March 5, 2026. The copyright holder for this preprint (which was not certified by peer review) is the author/funder, who has granted bioRxiv a license to display the preprint in perpetuity. It is made available under aCC-BY 4.0 International license.

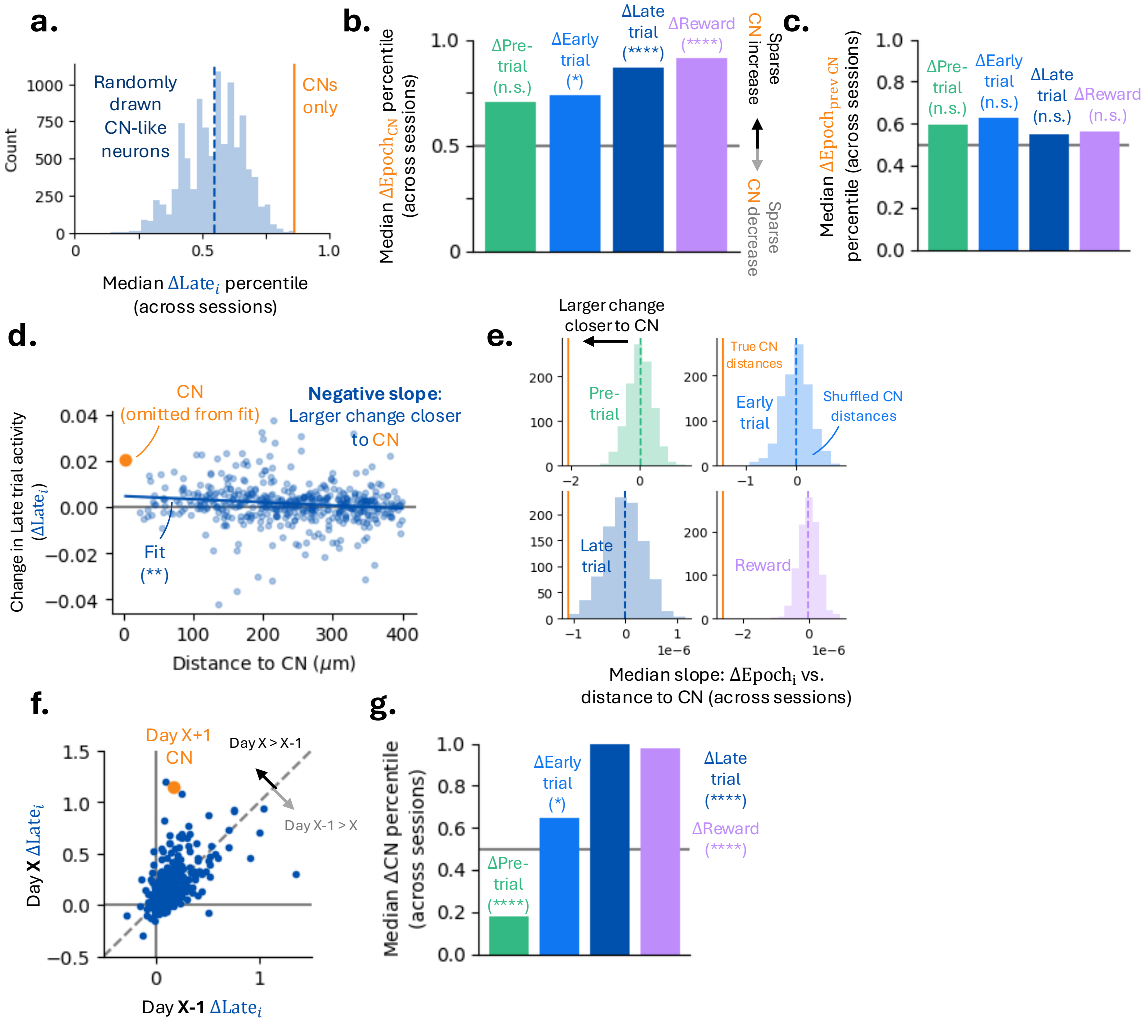

Figure S2: Supplement to Figure 2. **(a)** Same as Fig. 2f, but random neurons are only drawn from neurons that would be considered candidate CN neurons (Methods). **(b)** Same as Fig. 2g, with random neurons drawn from CN-like candidates only. From left to right, $p = 0.088, 0.088, < 10^{-4}, < 10^{-4}$. **(c)** Same as Fig. 2g, with the CN from the previous day instead of the current day. **(d)** Change in late trial activity versus distance to the CN for an exemplar session. Linear fit, omitting CN, shown. Distance to CN truncated to 400 μm to minimize edge of FoV contributions. **(e)** Distribution of median slope of ΔEPOCH versus distance to the CN across sessions (e.g. as shown in (d)), for all 4 epochs. Distribution comes from shuffling distances to CN across neurons (10^3 samples). Orange line shows median across unshuffled distance to CN. Unshuffled slopes consistently show negative trends, indicating larger changes closer to the CN. **(f)** Across session change of LATE TRIAL, showing values for each neuron on Day X and Day X+1. **(g)** Same as Fig. 2g, where across session change, shown in (f), is used instead of within session change.

bioRxiv preprint doi: https://doi.org/10.64898/2026.03.03.709199; this version posted March 5, 2026. The copyright holder for this preprint (which was not certified by peer review) is the author/funder, who has granted bioRxiv a license to display the preprint in perpetuity. It is made available under aCC-BY 4.0 International license.

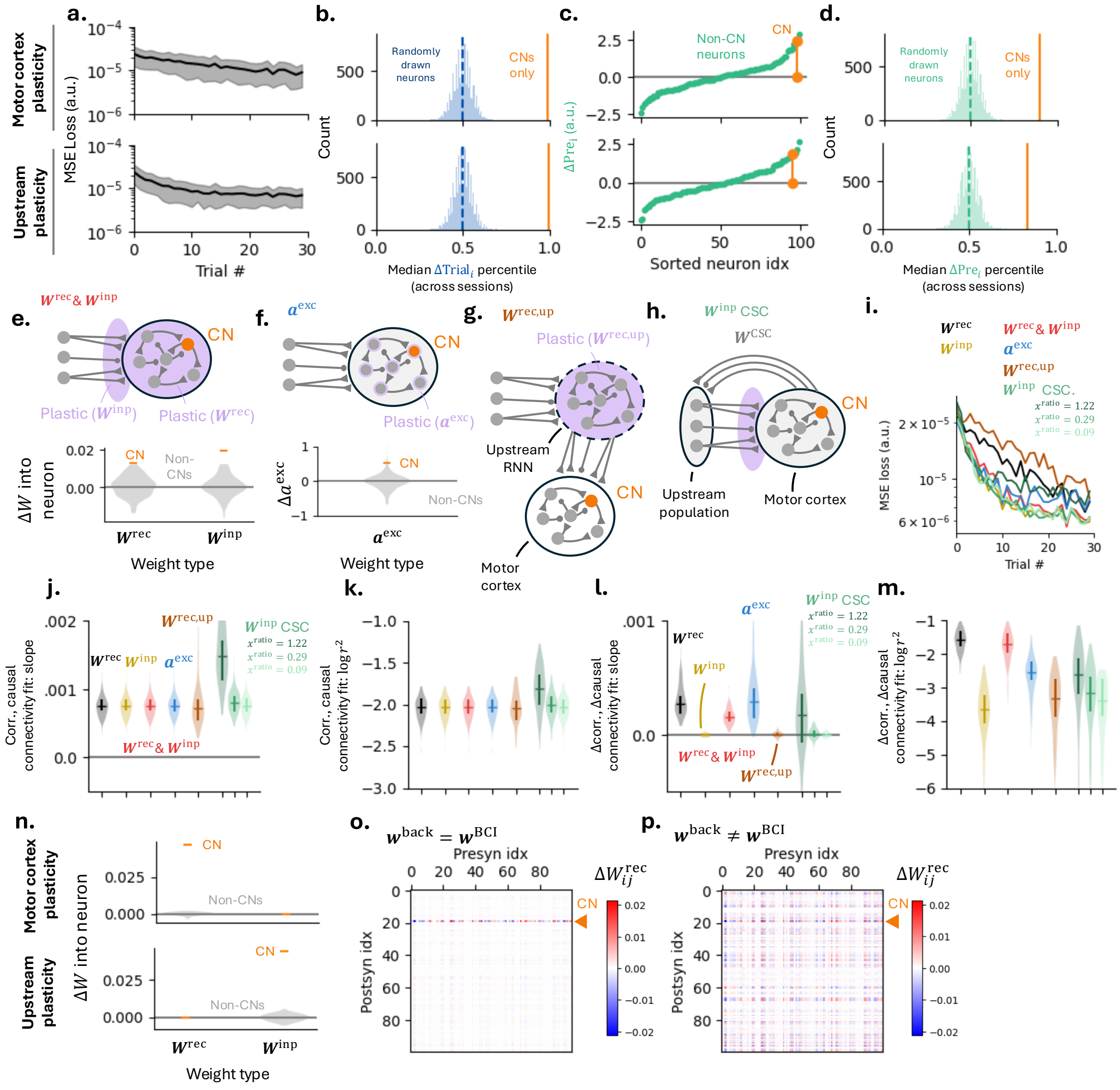

Figure S3: Supplement to Figure 3. **[a-d]** Additional results from local MC (top) and upstream (bottom) plasticity models. Aggregate results averaged over 100 separate initializations. **(a)** MSE loss versus trial # (mean with standard deviation shading). **(b)** Distribution of median ΔTRIAL percentile across all sessions for randomly sampled neurons from each session, 10^4 samples. Orange line shows median across the CN from all sessions. Model version of Fig. 2f. **(c)** Same as Fig. 3c, for PRETRIAL. **(d)** Same as (b), for PRETRIAL. **[e-m]** Alternative model comparisons. **(e)** Top: schematic of RNN model that learns via both MC and upstream plasticity ('$\mathbf{W}^{\text{rec}}$ & $\mathbf{W}^{\text{inp}}$'). Bottom: exemplar weight changes from training. **(f)** Top: schematic of excitability trained RNN ('$\mathbf{a}^{\text{exc}}$'). Bottom: exemplar excitability changes from training. **(g)** Schematic where upstream input is another RNN and its recurrent layer is trained ('$\mathbf{W}^{\text{rec, up}}$'). **(h)** Schematic of RNN with additional feedback from hidden layer to input layer, potentially representing cortical-subcortical loops ('$\mathbf{W}^{\text{inp}}$ CSC'). **(i)** Median loss functions across 100 initializations of each type of network. '$\mathbf{W}^{\text{inp}}$' and '$\mathbf{W}^{\text{rec}}$' are the upstream and MC plasticity models discussed in the main text. Distinct cortical-subcortical loop models shown for different connection strengths, quantified by $x^{\text{ratio}} > 0$, with larger values corresponding to stronger cortical-subcortical connections (Methods). **(j)** Correlation-causal connectivity fits performed analogously as Fig. 3f for each initialization. Distribution of slopes from fits across 100 initializations of each network type (dark line: median with 1st-3rd quartile bar). **(k)** Same as (j), but r^2 of fits. **(l)** Same as (j), but Δcorrelation-Δcausal connectivity fits, analogous to Fig. 3g. **(m)** Same as (l), but r^2 of fits. **[n-p]** Feedback misalignment of models (Methods). **(n)** Same as Fig. 3d, but for networks with feedback weights equal to BCI mask, $\mathbf{w}^{\text{back}} = \mathbf{w}^{\text{BCI}}$. **(o)** Exemplar $\Delta\mathbf{W}^{\text{rec}}$ over training for a network where $\mathbf{w}^{\text{back}} = \mathbf{w}^{\text{BCI}}$. Weights with postsynaptic CN indicated. **(p)** Same as (o), but where $\mathbf{w}^{\text{back}} \neq \mathbf{w}^{\text{BCI}}$, representative of models used in main text.

bioRxiv preprint doi: https://doi.org/10.64898/2026.03.03.709199; this version posted March 5, 2026. The copyright holder for this preprint (which was not certified by peer review) is the author/funder, who has granted bioRxiv a license to display the preprint in perpetuity. It is made available under aCC-BY 4.0 International license.

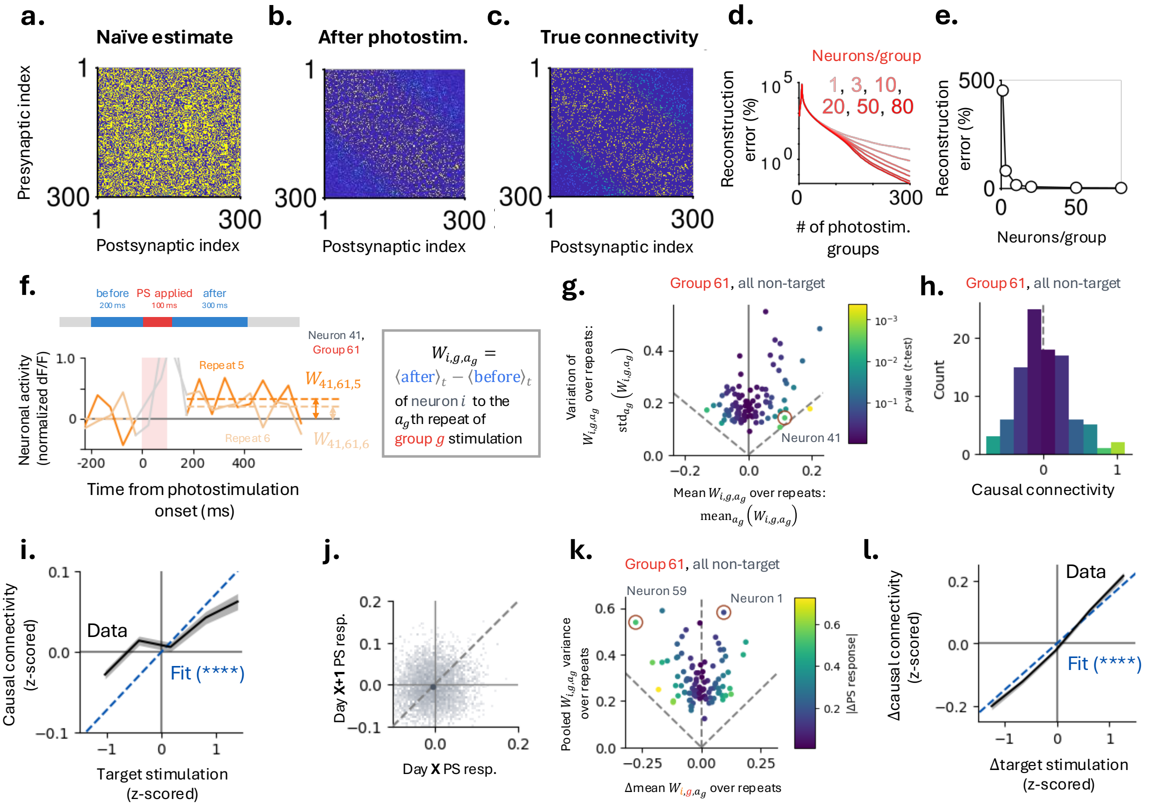

Figure S4: Supplement to Figure 4. [a-e] Photostimulation reconstruction versus number of stimulated sites. **(a)** Estimate of reconstructed connectivity from spontaneous activity. **(b)** Same as (a), from spontaneous activity and 300 photostimuli with 80 neurons per photostimulation group. **(c)** True connectivity of estimates in (a) and (b). **(d)** Reconstruction error versus number of distinct photostimulation groups for $1, 3, 10, 20$, and 80 neurons per stimulation group. **(e)** Reconstruction error versus number of neurons per group, for 300 photostimulation groups. **[f-i]** Causal connectivity. **(f)** Change in activity exemplar non-target neuron for two photostimulation repeats of same group (left). Definition of repeat response, which quantifies the photostimulation response to a given stimulation (right). **(g)** Mean repeat response versus standard deviation of repeat response, over repeats, for all non-target neurons of a given photostimulation group. Neurons colored by significance of response (t-test). **(h)** Causal connectivity of neurons shown in (g), colored by mean significance, same scale as (g). **(i)** Total target photostimulation versus non-target causal connectivity. **[j-l]** Change in causal connectivity. **(j)** Same as top of Fig. 4f, for non-target neurons. **(k)** Change in mean repeat response versus pooled repeat response variances, see Eq. (4). Neurons colored by Δcausal connectivity. **(l)** Change in total target photostimulation versus non-target Δcausal connectivity.

bioRxiv preprint doi: https://doi.org/10.64898/2026.03.03.709199; this version posted March 5, 2026. The copyright holder for this preprint (which was not certified by peer review) is the author/funder, who has granted bioRxiv a license to display the preprint in perpetuity. It is made available under aCC-BY 4.0 International license.

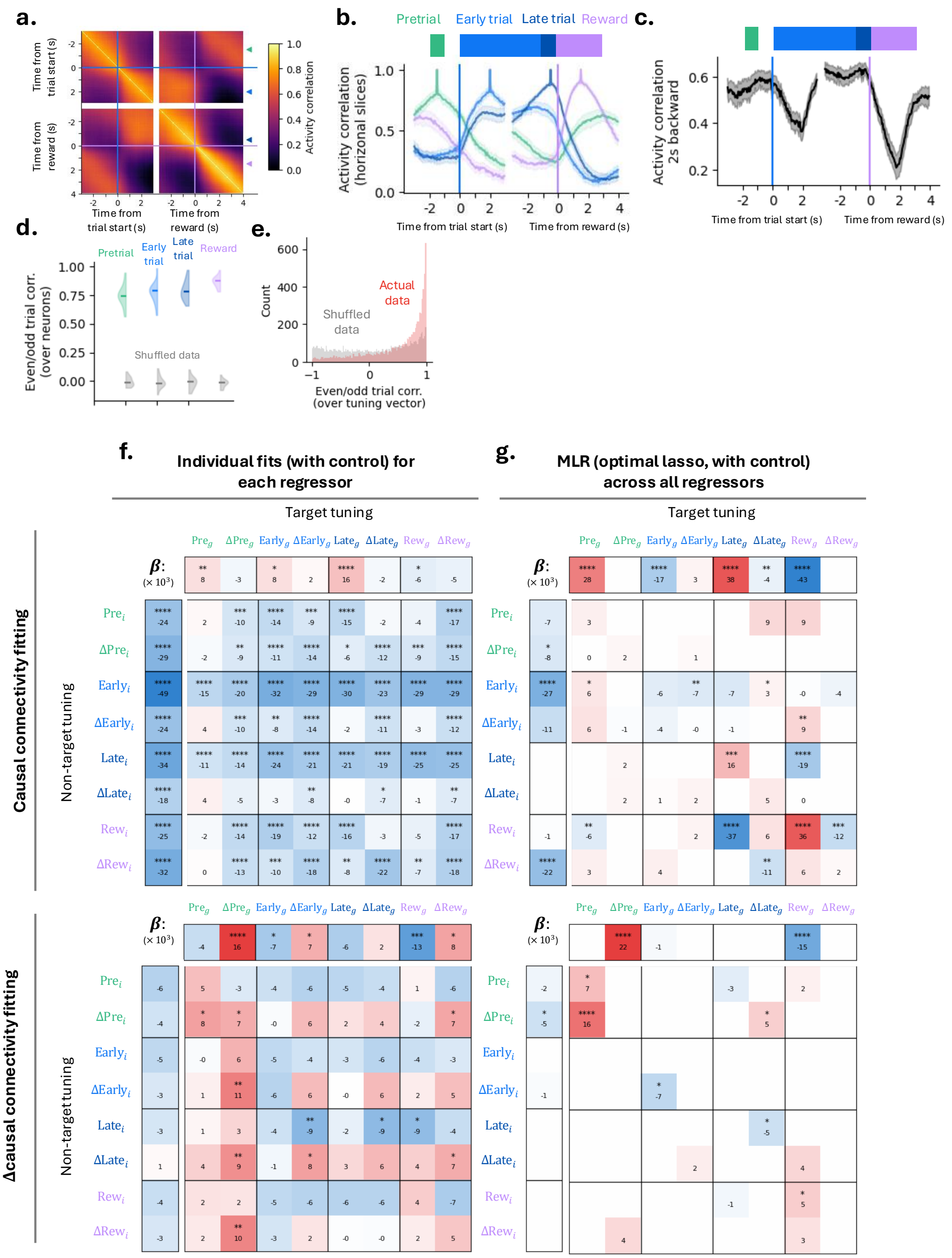

Figure S5: Supplement to Figure 5. **(a)** Correlation of activity across task epochs, averaged across sessions. Horizontal slices shown in (b) denoted by triangles on right side. **(b)** Horizontal slices of similarity for exemplar times within each of the task epochs. **(c)** Activity correlation of current and 2 seconds backward showing variation in population activity versus time. **(d)** Correlation of neurons' tuning when computed on only even/odd trials, computed over neurons for each task epoch. Grey shows data shuffled over neurons, solid line shows median over sessions. **(e)** Correlation of the tuning vector for each neuron when computed on only even/odd trials, computed over tack epochs for each neuron. **(f)** Fitting of causal connectivity (top) and Δcausal connectivity (bottom) using each individual regressor used in Figs. 5, 6. Unlike the MLR fits used in Figs. 5, 6, each fit only consists of the corresponding regressor and the control (Methods). **(g)** MLR fitting of causal connectivity (top) and Δcausal connectivity (bottom) using the eight-dimensional task tuning vectors of Fig. 6 (i.e. top is generalization of Fig. 5i, bottom is same as Fig. 6c for easy comparison).

bioRxiv preprint doi: https://doi.org/10.64898/2026.03.03.709199; this version posted March 5, 2026. The copyright holder for this preprint (which was not certified by peer review) is the author/funder, who has granted bioRxiv a license to display the preprint in perpetuity. It is made available under aCC-BY 4.0 International license.

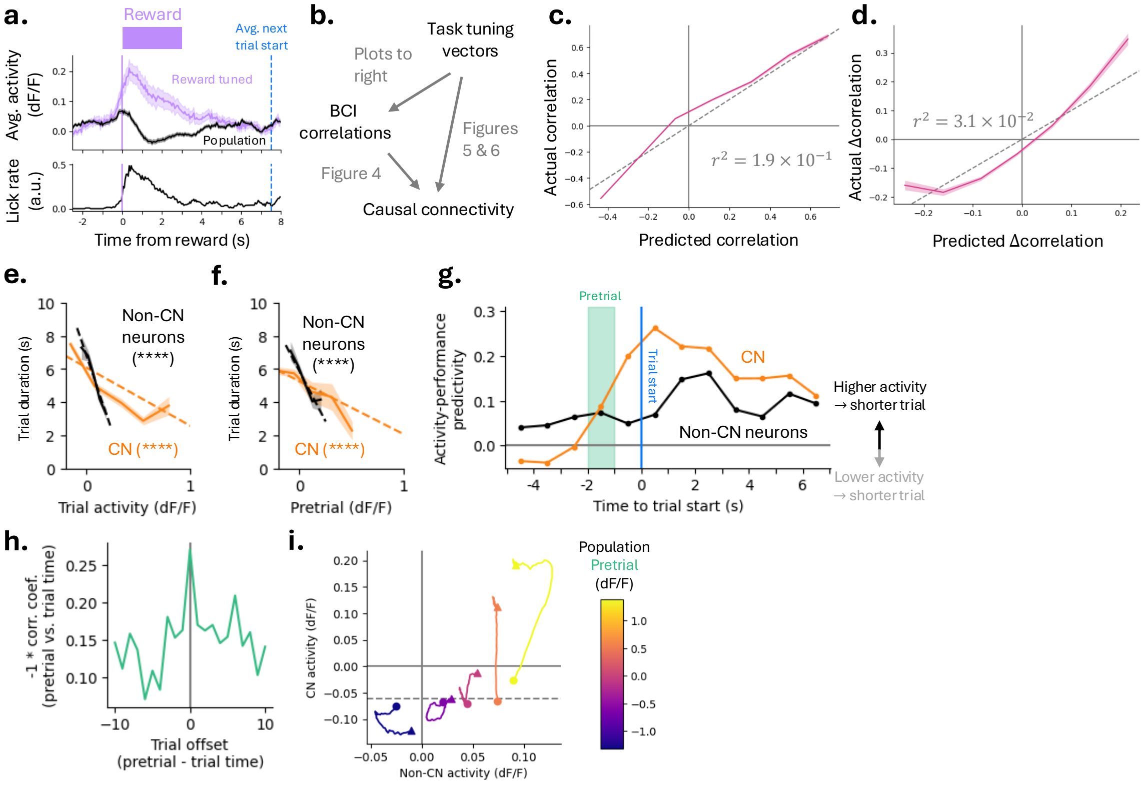

Figure S6: Supplement to Figure 6. (a) Mean reward-aligned activity for reward responsive neurons and full population activity (top) and lick rate (bottom) as a function of time from reward. Shows population activity is modulated during reward consumption. **(b)** Relationship between the various routes of explaining causal connectivity and correlations of activity during BCI task used throughout the text. **(c)** MLR model fitting BCI correlations, uses same regressors as those determined from cross-validated lasso in Fig. 5i. **(d)** MLR model fitting ΔBCI correlations, uses same regressors as those determined from cross-validated lasso in Fig. 6c. **(e)** Trial duration as a function the CN (orange) and mean non-CN (black) trial activity, showing higher trial activity leads to shorter trial durations. Trial activity defined as the mean activity over entire trial (until timeout or reward). Solid lines show mean binned data, with s.e.m. shading. Dotted lines are linear regression fits. **(f)** Same as (e), but now as a function of PRETRIAL activity. **(g)** Activity-performance predictivity, defined as the –sgn(slope) $* r^2$ of the linear regression fits shown in (e) and (f), for 1 second bins aligned to trial start. Shows predictivity increases within trial, as expected, but remains high directly prior to trial start as well. PRETRIAL shaded region corresponds to dotted lines in (f). **(h)** How correlation of PRETRIAL activity and trial duration changes as PRETRIAL activity and trial duration are offset from one another. Shows correlation magnitude is highest for PRETRIAL directly prior to said trial (i.e. 0 offset). **(i)** Evolution of non-CN activity and CN activity from low-CN time point to trial start, same binning shown in Fig. 6e.

bioRxiv preprint doi: https://doi.org/10.64898/2026.03.03.709199; this version posted March 5, 2026. The copyright holder for this preprint (which was not certified by peer review) is the author/funder, who has granted bioRxiv a license to display the preprint in perpetuity. It is made available under aCC-BY 4.0 International license.

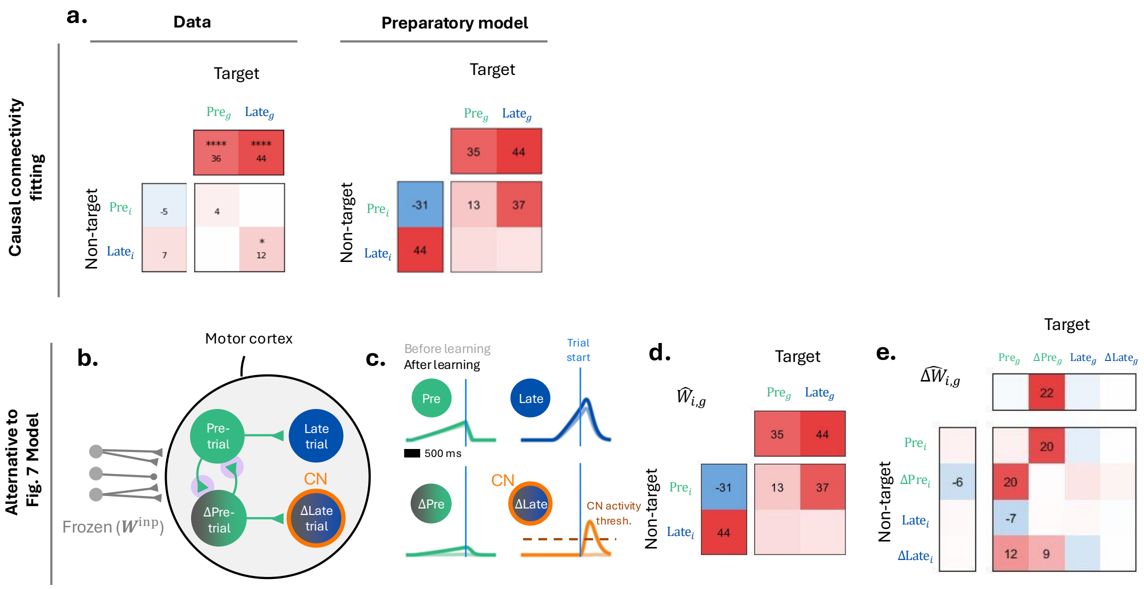

Figure S7: Supplement to Figure 7. (a) Left: Subset of MLR coefficients fitting causal connectivity, shown in Fig. 5h, to be matched by the model. Right: MLR coefficients fitting model causal connectivity, indicating which task-tuning features predict connectivity (colored by size of coefficient, a.u.). **[b-e]** Same as Fig. 7, for an alternative weight change configuration. **(b)** Schematic diagram of the preparatory network. Magenta circles indicate new connections that are added during learning. **(c)** Activity of PRETRIAL, ΔPRETRIAL, LATE TRIAL, and ΔLATE TRIAL neurons before (light) and after (dark) learning. **(d)** Same as (a, right), for alternative weight change configuration. **(e)** Δcausal connectivity matrix calculated using MLR as in Fig. 6c.
